# Supplementary material for: Recurrent rearrangements of FOS and FOSB define osteoblastoma
Source: Nat Commun. 2018 Jun 1;9:2150. doi: 10.1038/s41467-018-04530-z (PMC5984627; doi:10.1038/s41467-018-04530-z)

Recurrent rearrangements of *FOS* and *FOSB* define osteoblastoma

Supplementary Figures

Fittall *et al*

Supplementary Figure 1. Copy number profiles for the sequenced osteoblastoma samples
a) Genome wide views. Copy number is shown on the y-axis and genomic position on the x-axis. Total copy number is shown as purple segments and the minor copy number state is shown in orange.
b) Detailed analysis of chromosome 22. Total copy number is shown in green segments, minor allele copy number is in green in the top panel. Sequentially below plots reveal LogR (ratio of tumour to normal coverage as computed by ASCAT), tumour coverage, normal sample coverage, B-Allele Frequency (BAF). In LogR and BAF plots, poorly performing snps are highlighted in red. No reliable copy number abberrations are seen across chromosome 22 in any sample.

a

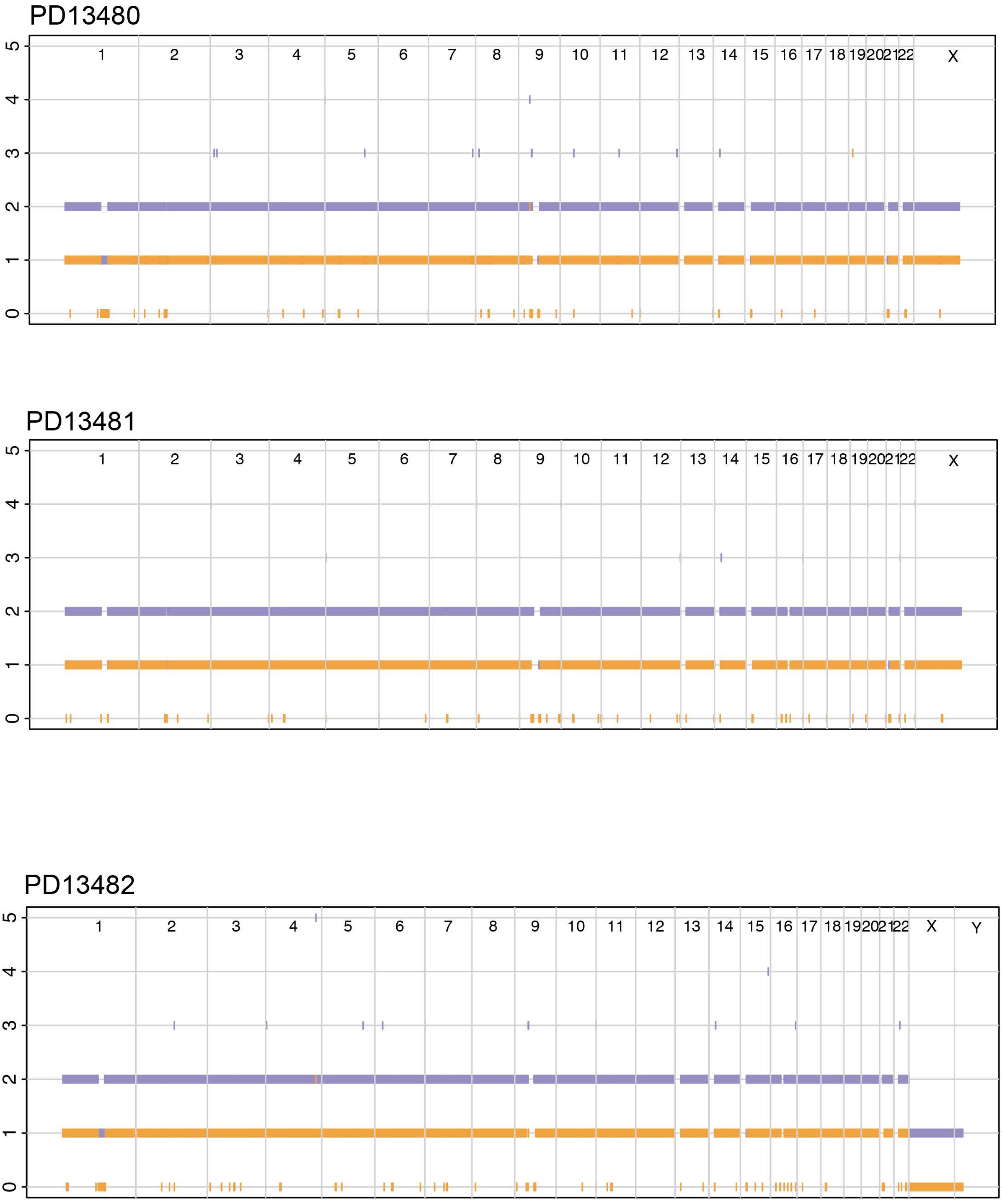

PD7519

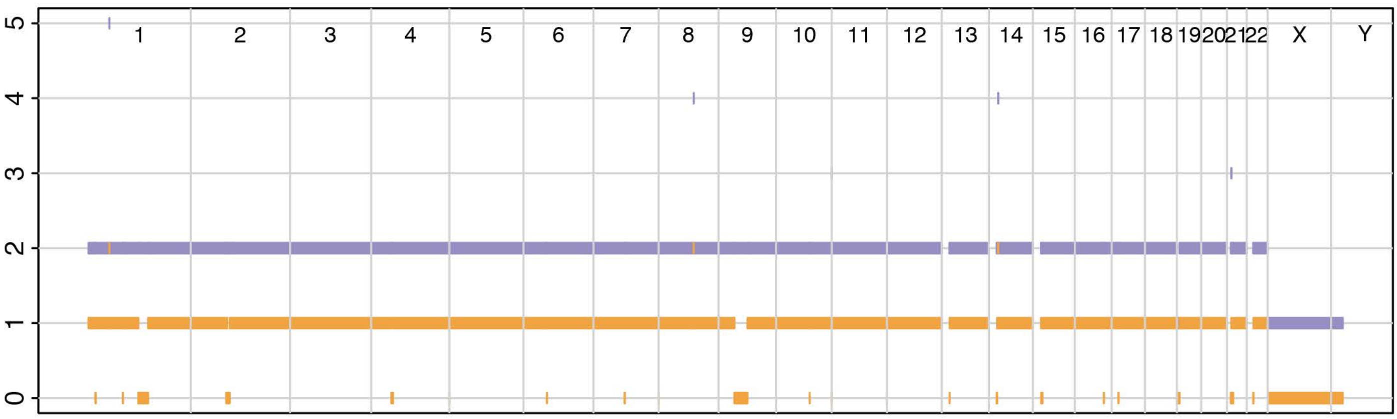

PD7521

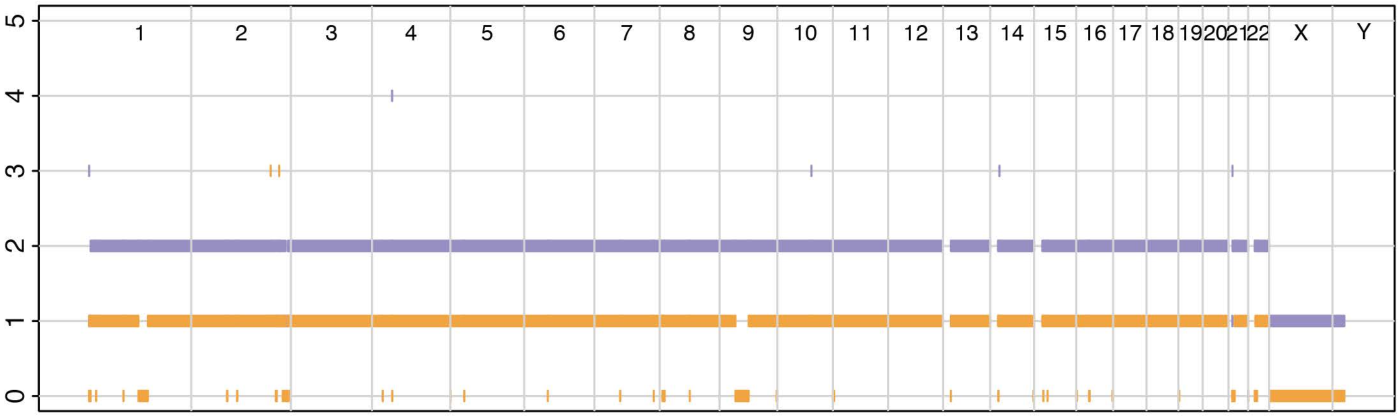

PD7525

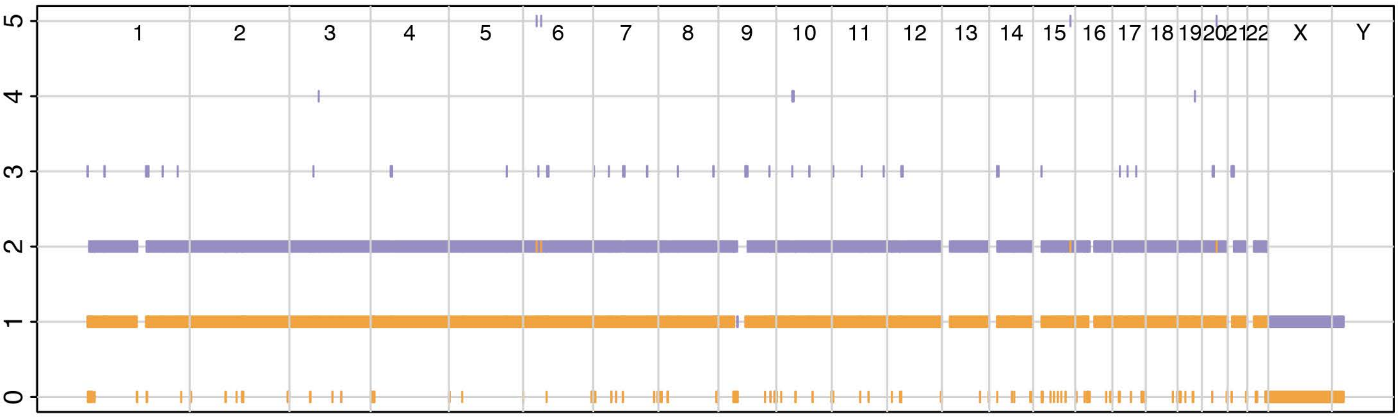

b PD13480

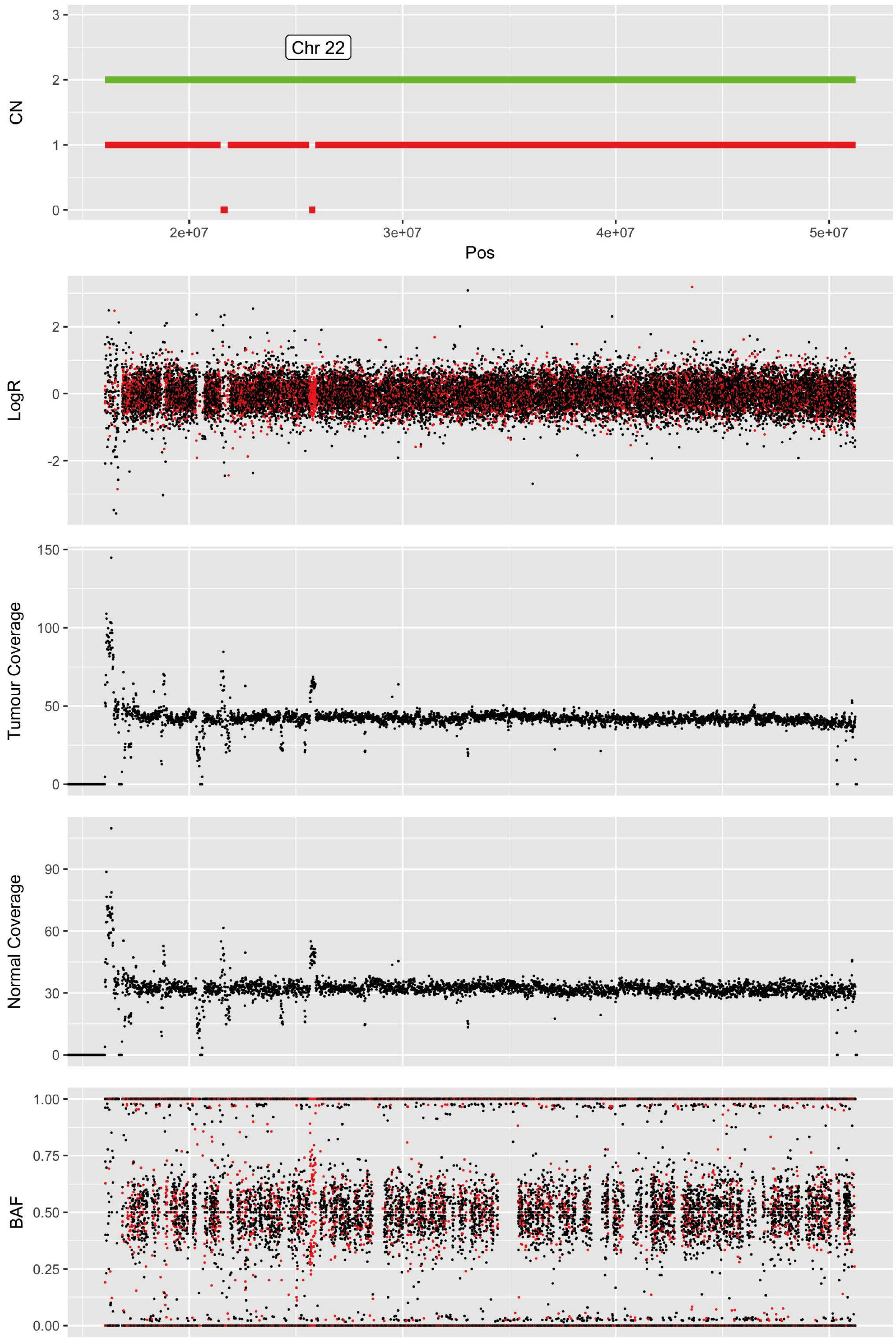

Chr 22
CN
3
2
1
0
2e+07
3e+07
4e+07
5e+07
Pos
LogR
2
0
-2
Tumour Coverage
150
100
50
0
Normal Coverage
90
60
30
0
BAF
1.00
0.75
0.50
0.25
0.00

PD13481

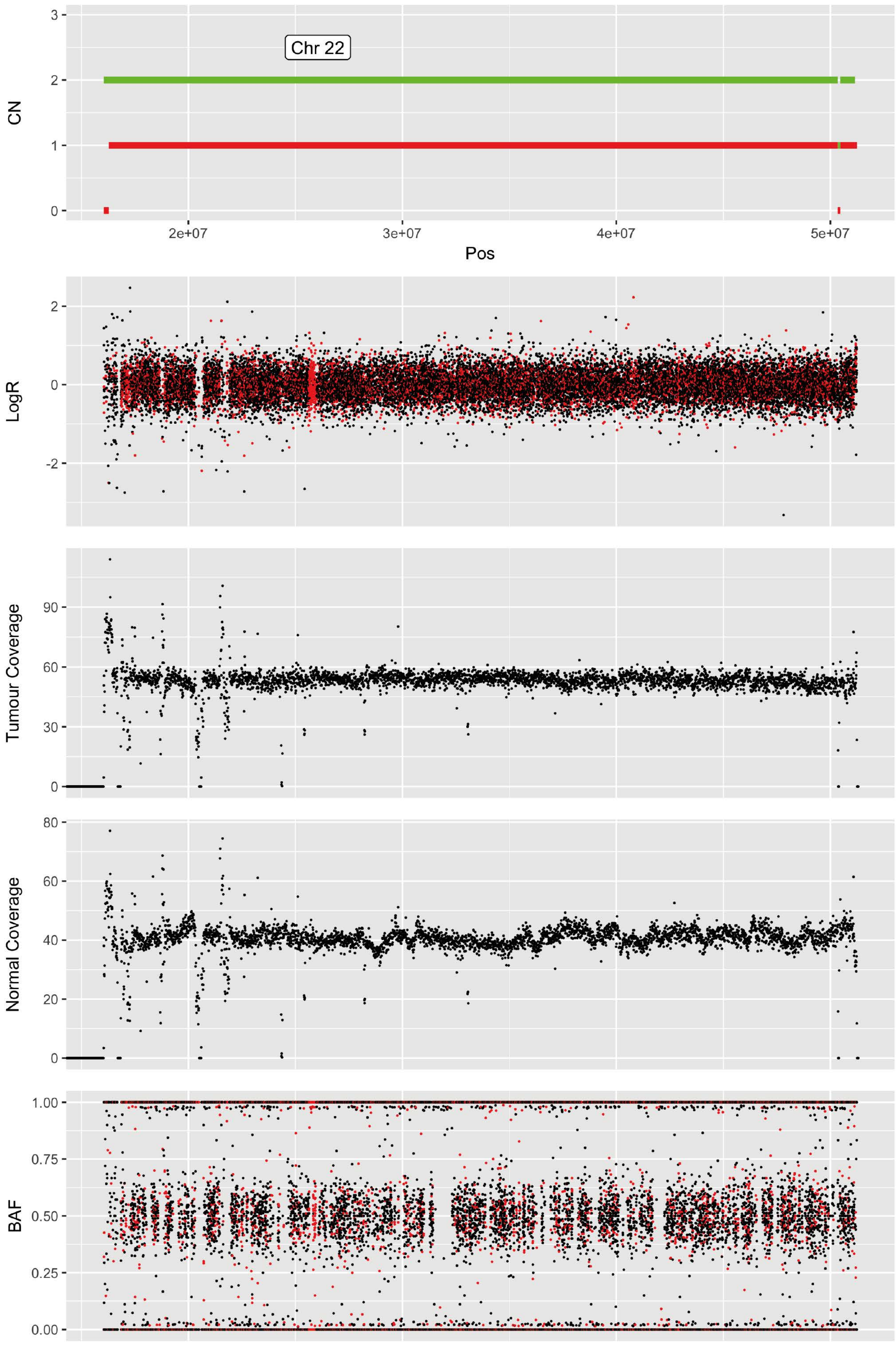

Chr 22
CN
3
2
1
0
2e+07
3e+07
4e+07
5e+07
Pos
LogR
2
0
-2
Tumour Coverage
90
60
30
0
Normal Coverage
80
60
40
20
0
BAF
1.00
0.75
0.50
0.25
0.00

PD13482

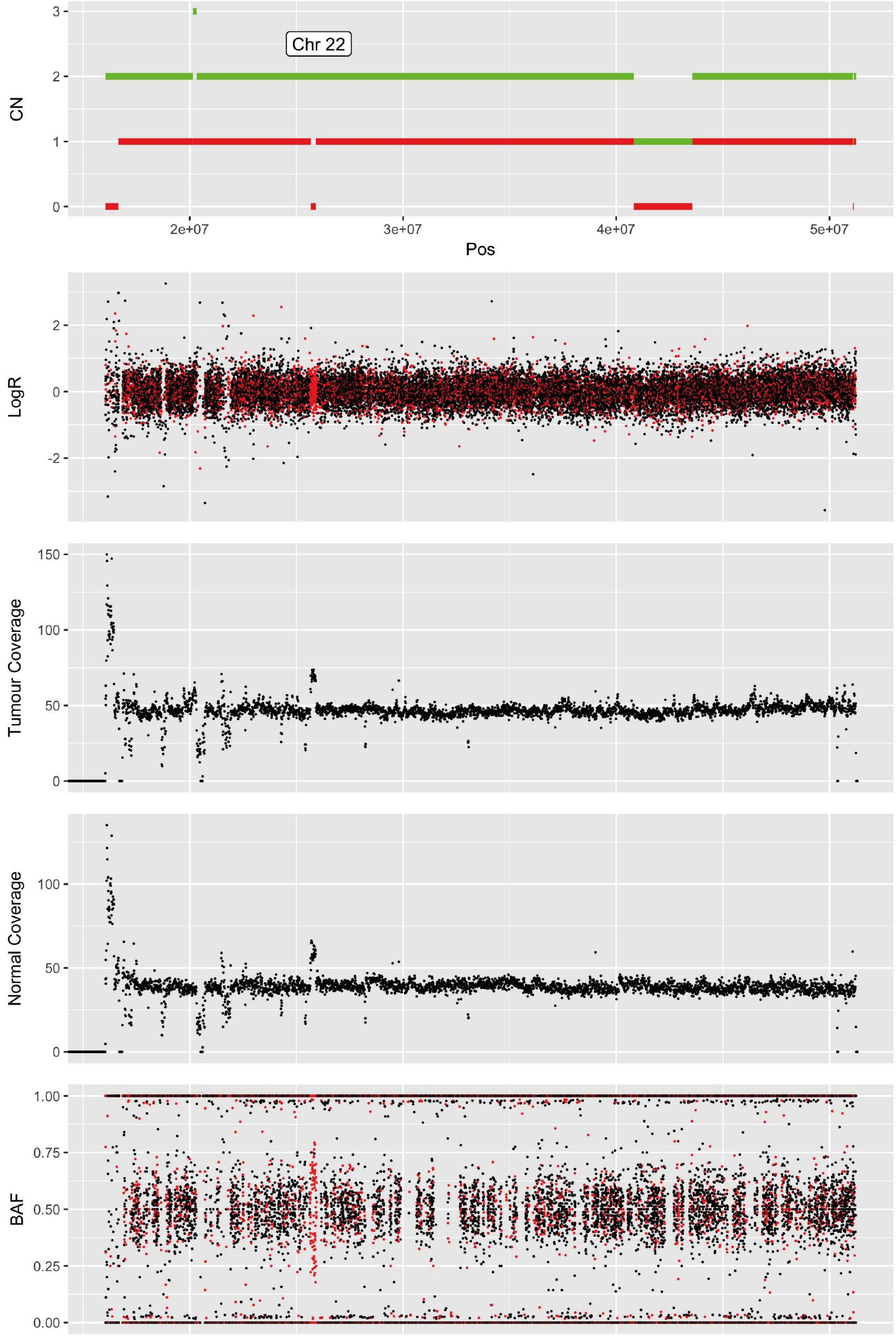

Chr 22
CN
3
2
1
0
2e+07
3e+07
4e+07
5e+07
Pos
LogR
2
0
-2
Tumour Coverage
150
100
50
0
Normal Coverage
100
50
0
BAF
1.00
0.75
0.50
0.25
0.00

PD7519

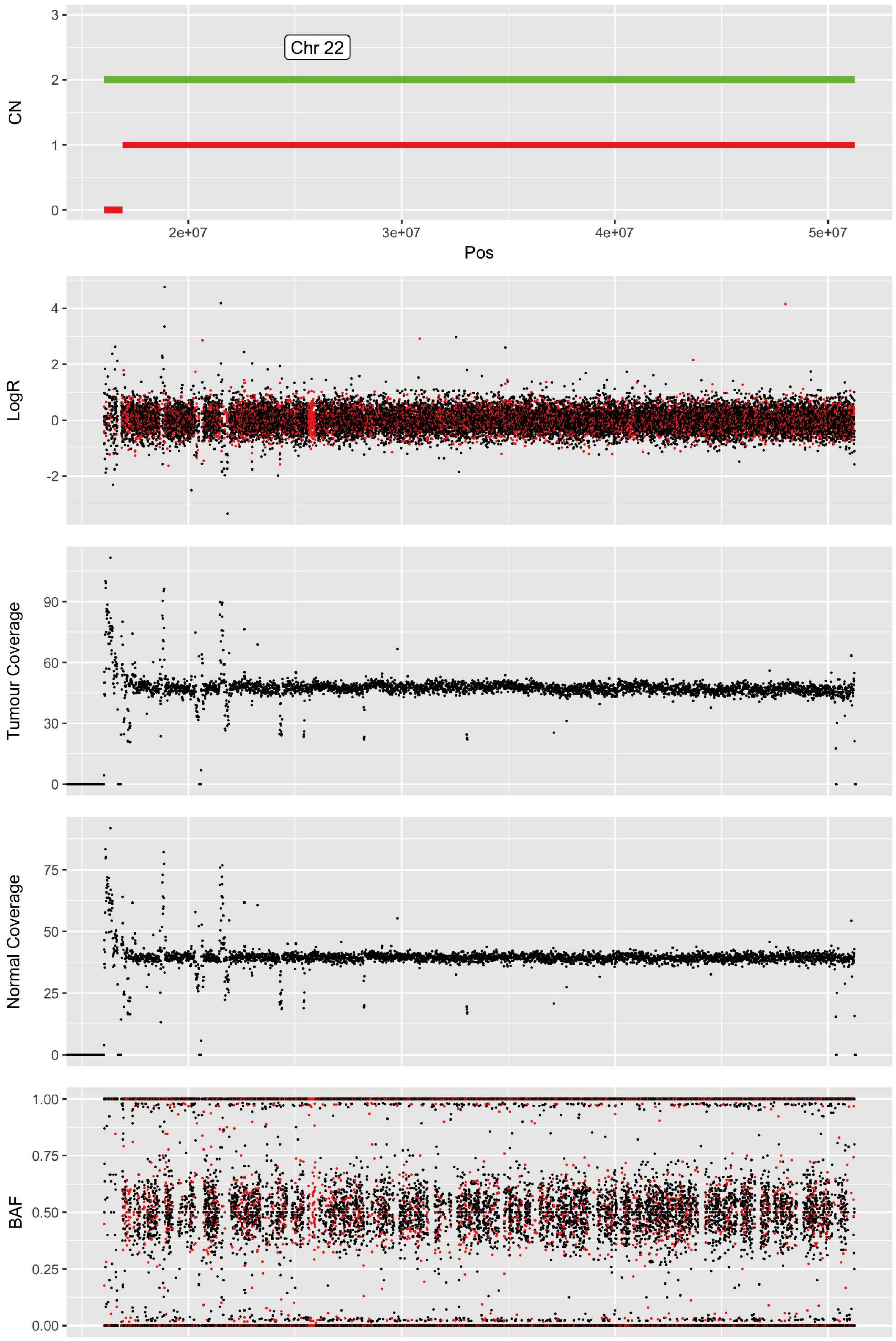
Chr 22
CN
3
2
1
0
2e+07
3e+07
4e+07
5e+07
Pos
LogR
4
2
0
-2
Tumour Coverage
90
60
30
0
Normal Coverage
75
50
25
0
BAF
1.00
0.75
0.50
0.25
0.00

PD7521

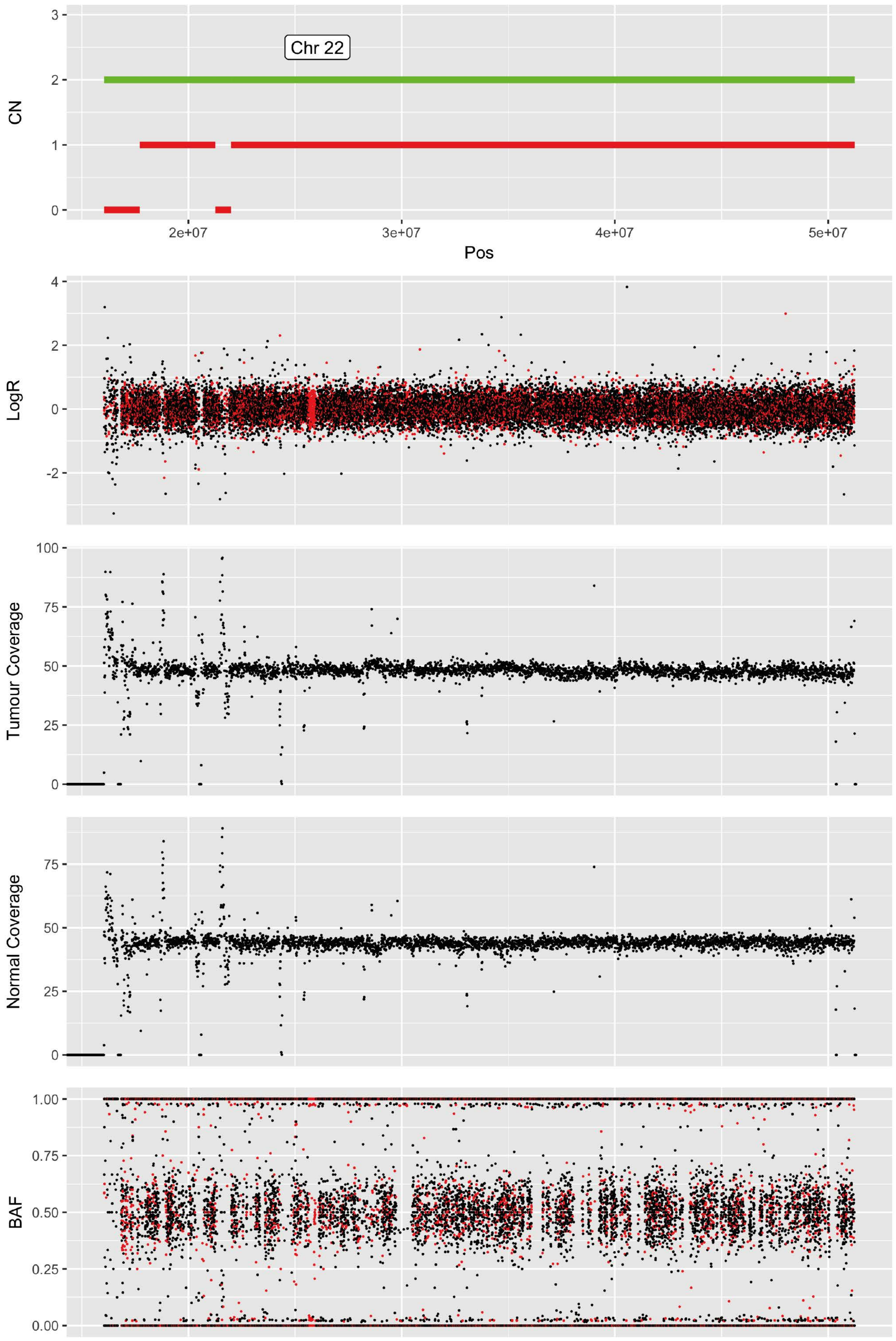

Chr 22
CN
3
2
1
0
2e+07
3e+07
4e+07
5e+07
Pos
LogR
4
2
0
-2
Tumour Coverage
100
75
50
25
0
Normal Coverage
75
50
25
0
BAF
1.00
0.75
0.50
0.25
0.00

PD7525

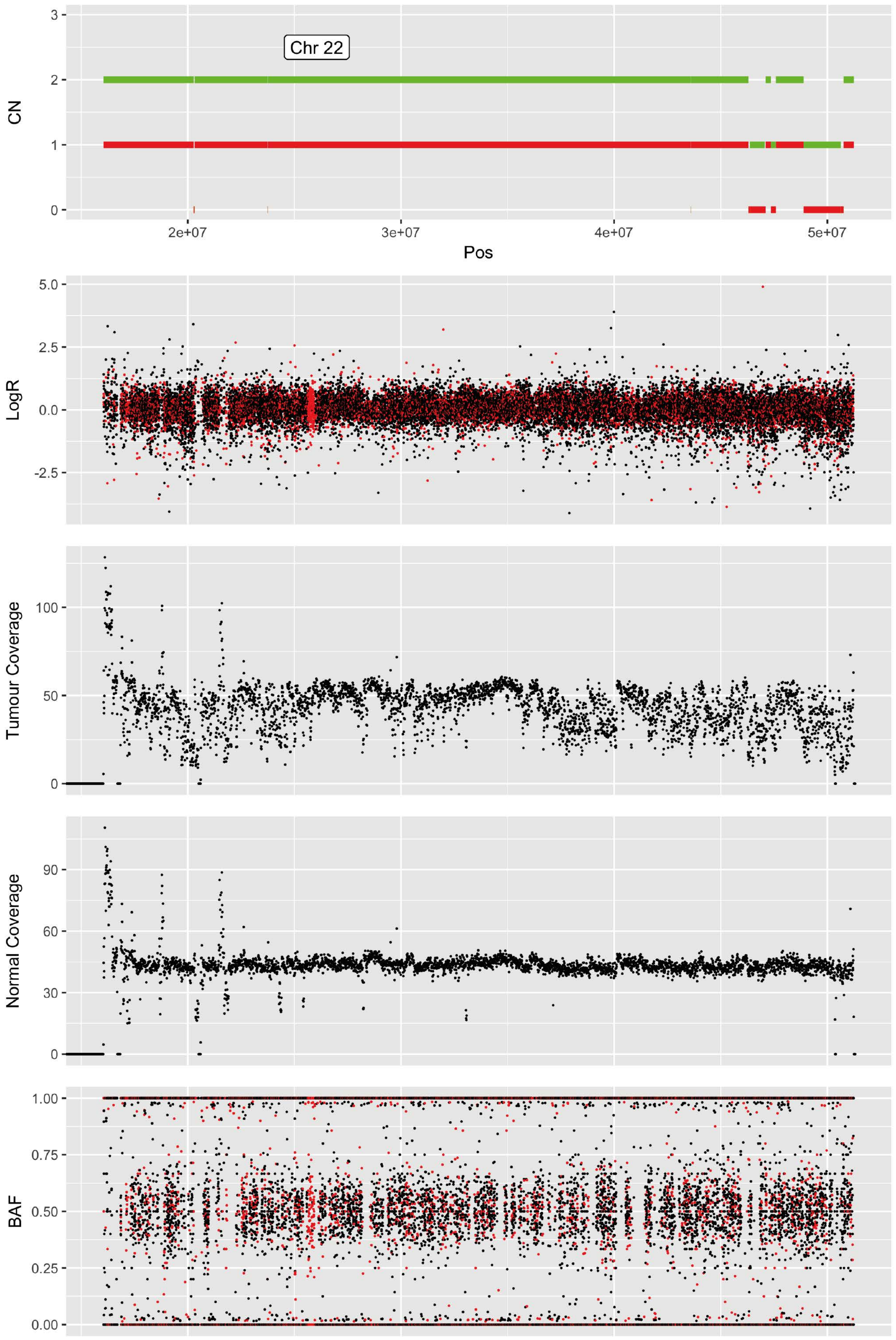
Chr 22
CN
3
2
1
0
2e+07
3e+07
4e+07
5e+07
Pos
LogR
5.0
2.5
0.0
-2.5
Tumour Coverage
100
50
0
Normal Coverage
90
60
30
0
BAF
1.00
0.75
0.50
0.25
0.00

Supplementary Figure 2. Fusion sequneces and Sanger sequencing validation of *FOS* fusions
Upper schematics show the cDNA sequence revealed from bulk sequencing reads with the predicted peptide sequence. Beneath is validation of the fusion sequence from Sanger sequencing across the (*FOS* fusions only).

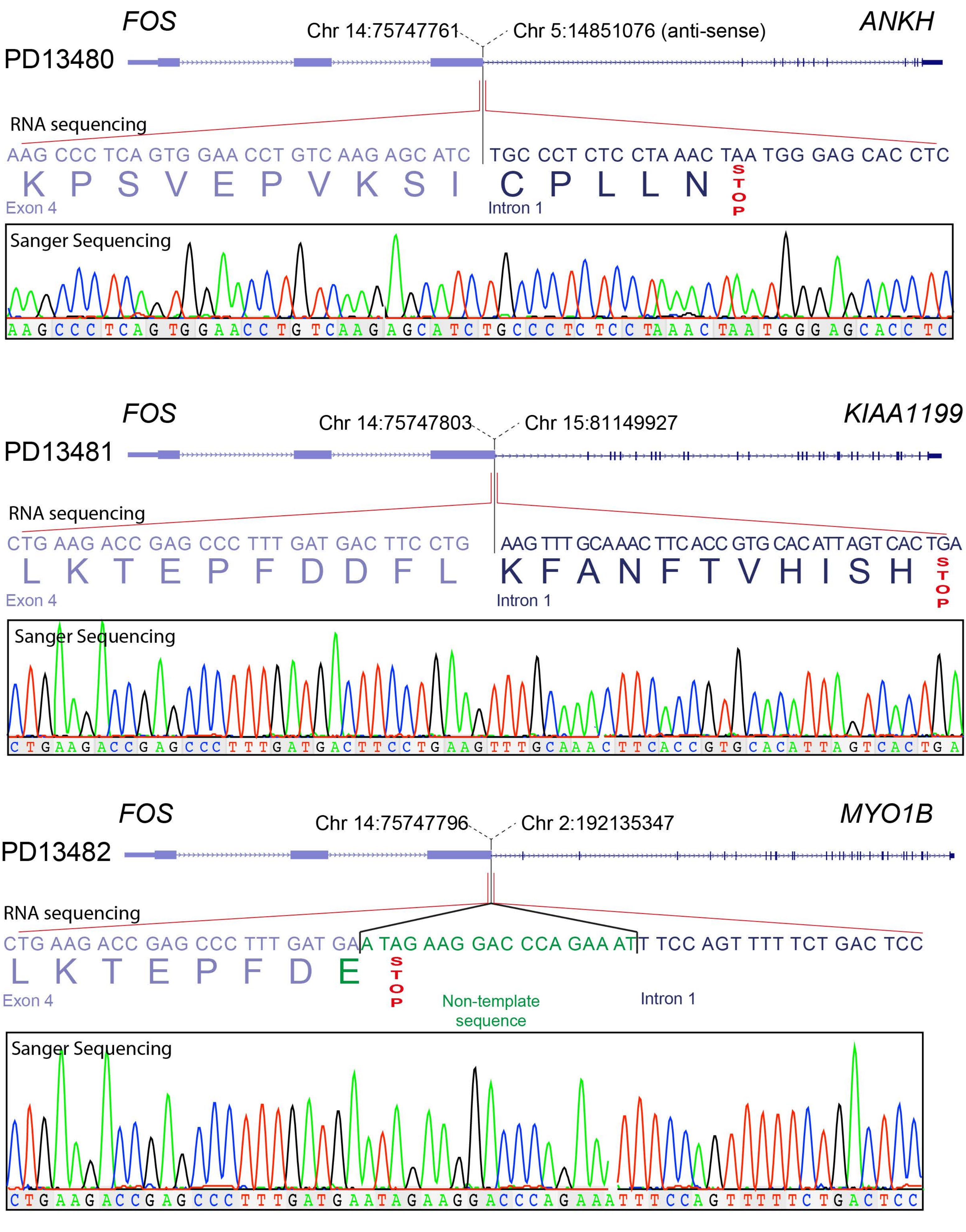

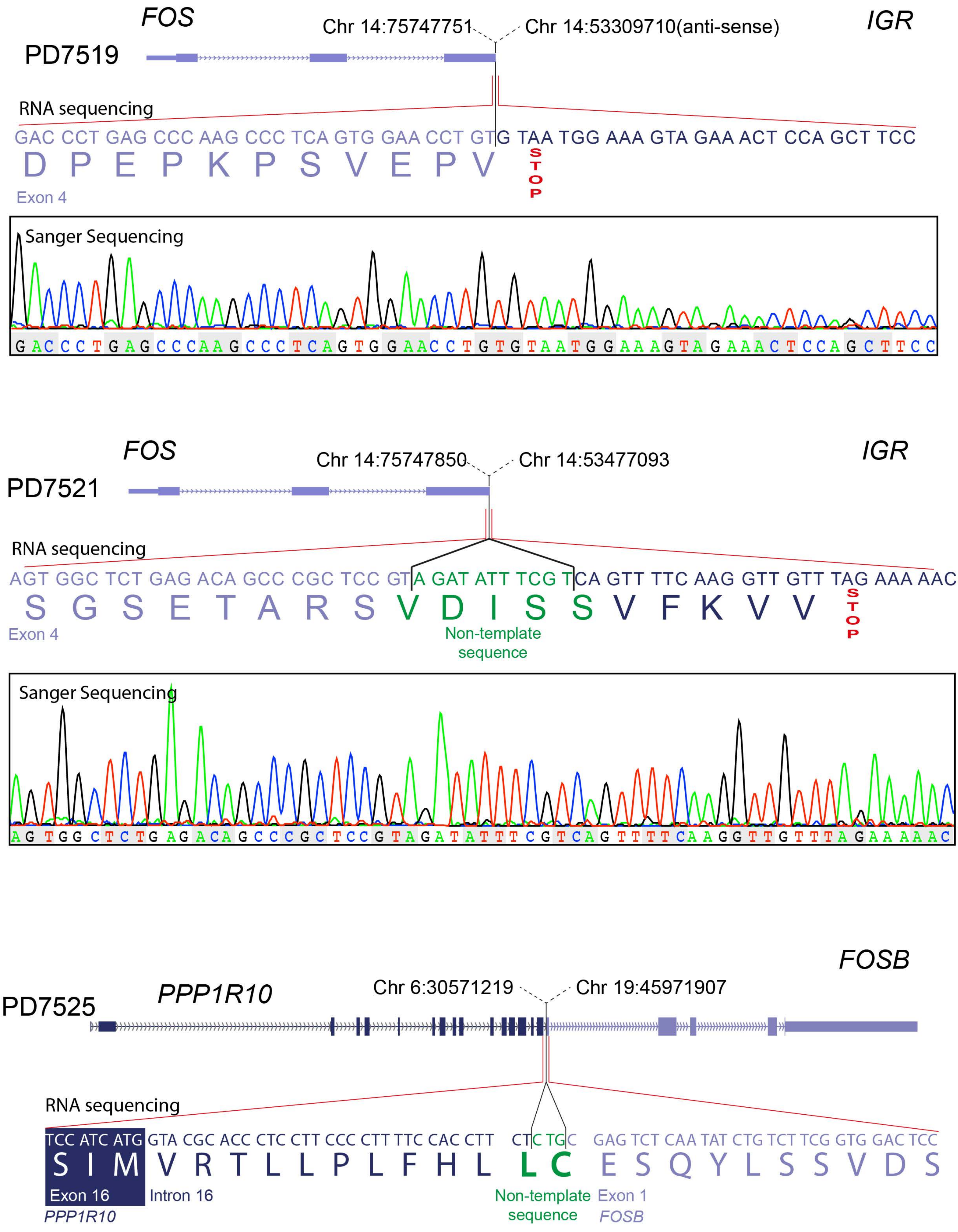

FOS
PD7519
Chr 14:75747751
Chr 14:53309710(anti-sense)
IGR
RNA sequencing
GAC CCT GAG CCC AAG CCC TCA GTG GAA CCT GTG TAA TGG AAA GTA GAA ACT CCA GCT TCC
D P E P K P S V E P V
STOP
Exon 4
Sanger Sequencing
FOS
PD7521
Chr 14:75747850
Chr 14:53477093
IGR
RNA sequencing
AGT GGC TCT GAG ACA GCC CGC TCC GTA GAT ATT TCG TCA GTT TTC AAG GTT GTT TAG AAA AAC
S G S E T A R S V D I S S V F K V V
STOP
Exon 4
Non-template sequence
Sanger Sequencing
FOSB
PD7525
PPP1R10
Chr 6:30571219
Chr 19:45971907
RNA sequencing
TCC ATC ATG GTA CGC ACC CTC CTT CCC CTT TTC CAC CTT CTC TGC GAG TCT CAA TAT CTG TCT TCG GTG GAC TCC
S I M V R T L L P L F H L L C E S Q Y L S S V D S
Exon 16
PPP1R10
Intron 16
Non-template sequence
Exon 1
FOSB

Supplementary Figure 3. RNA sequencing coverage and allelic imbalance for *FOS* and *FOSB*

Plots show RNAseq coverage with exons shown as blue rectangles. Breakpoints are shown with red lines. Fusion partner coverage is shown for 1kb after the breakpoint (light blue). Heterozygous SNPs, as identified in DNA, relative counts are shown as stacked bars.a-e) *FOS* fusion cases, f) *FOSB* fusion. There is no clear evidence of allelic imbalance or fusion transcripts dominating wild-type transcripts. As tumour purity is low, it is likely that a significant proportion of RNAseq reads are contributed by normal contaminating cells. As these are not immunoreactive on FOS immunostaining (see panels above or PD7525 for FOS fusion negative sample), this strongly hints at predominantly post-transcriptionally effects of truncation. g) For this FOSB fusion case there is imbalance of an intron 1 heterozygous snp, suggesting increased trasncriptional activity of one allele.

a

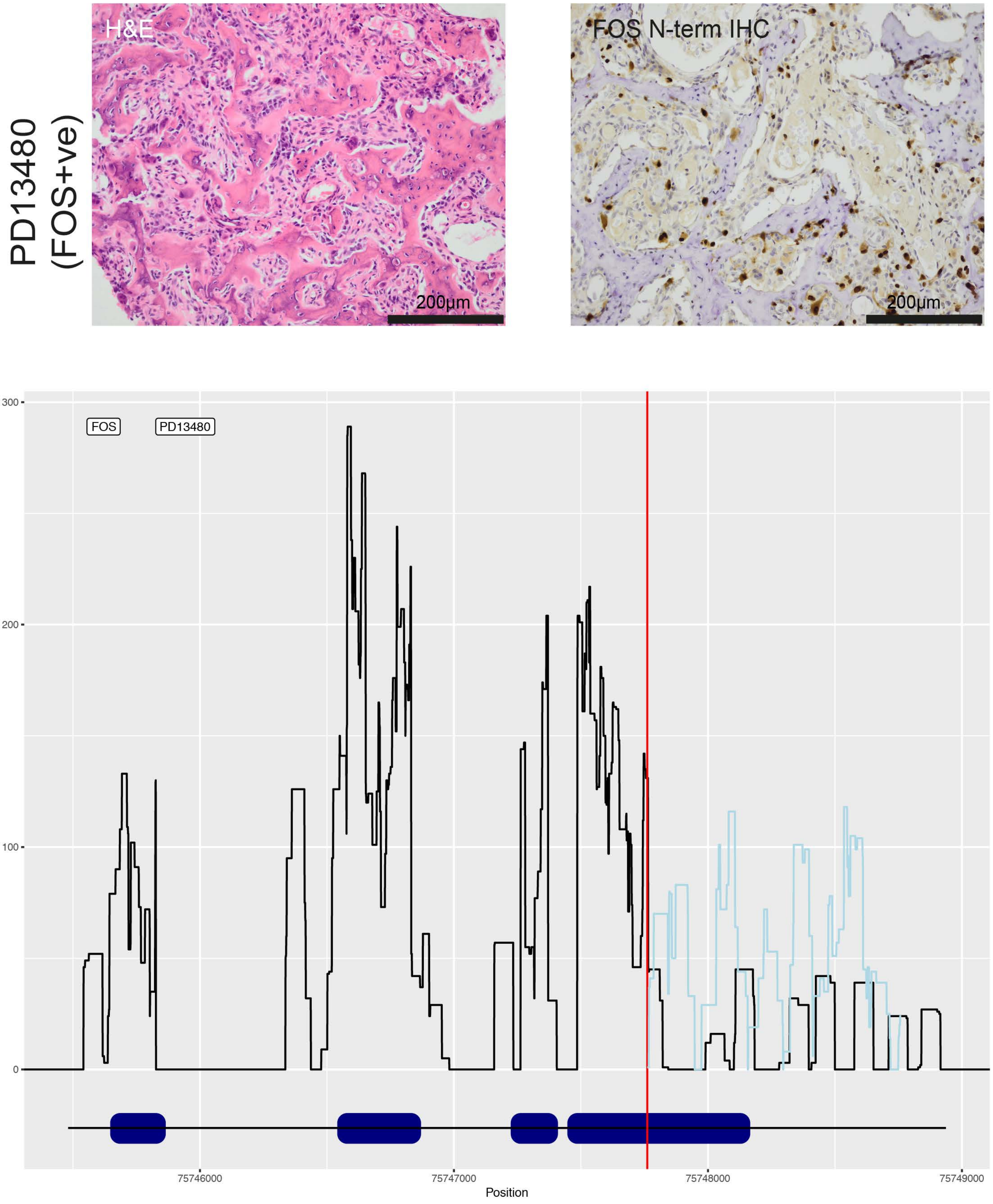

b

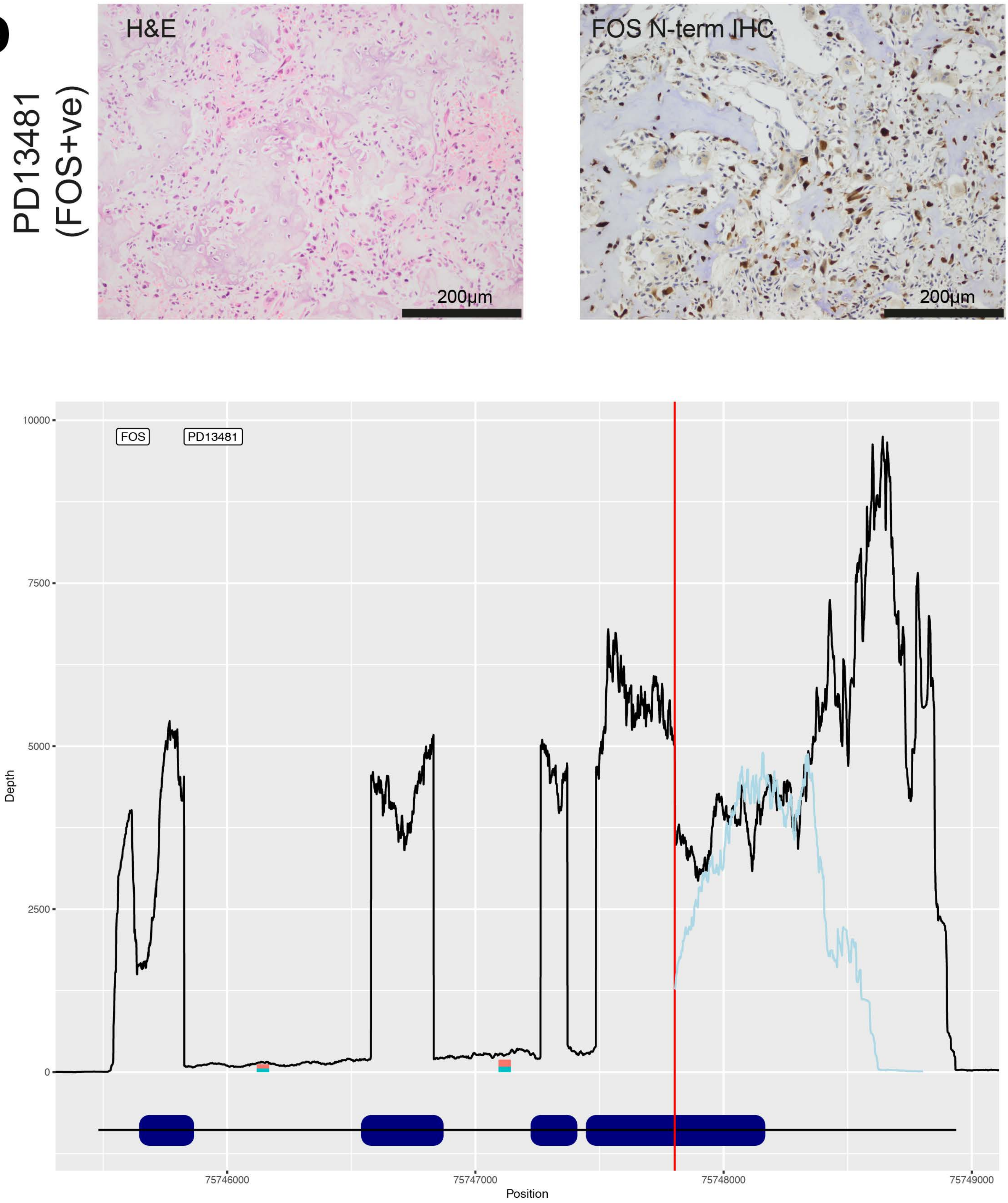

PD13481
(FOS+ve)
H&E
200μm
FOS N-term IHC
200μm
FOS
PD13481
Depth
10000
7500
5000
2500
0
75746000
75747000
75748000
75749000
Position

c

PD13482 (FOS+ve)

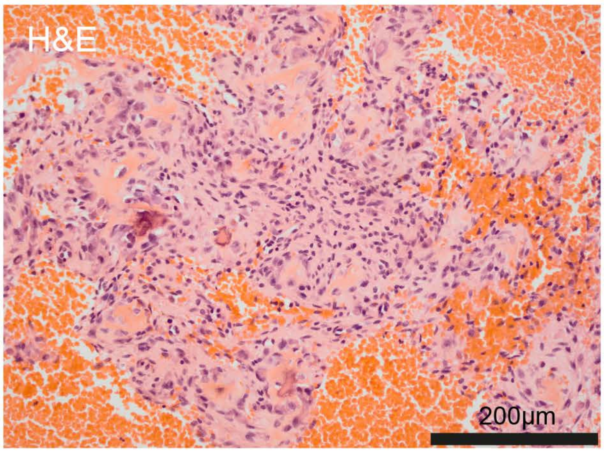

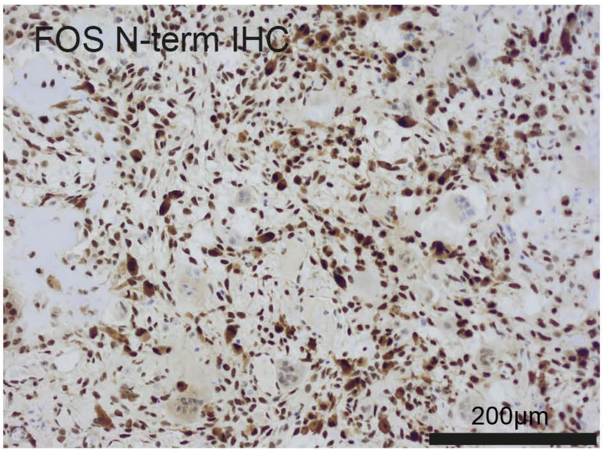

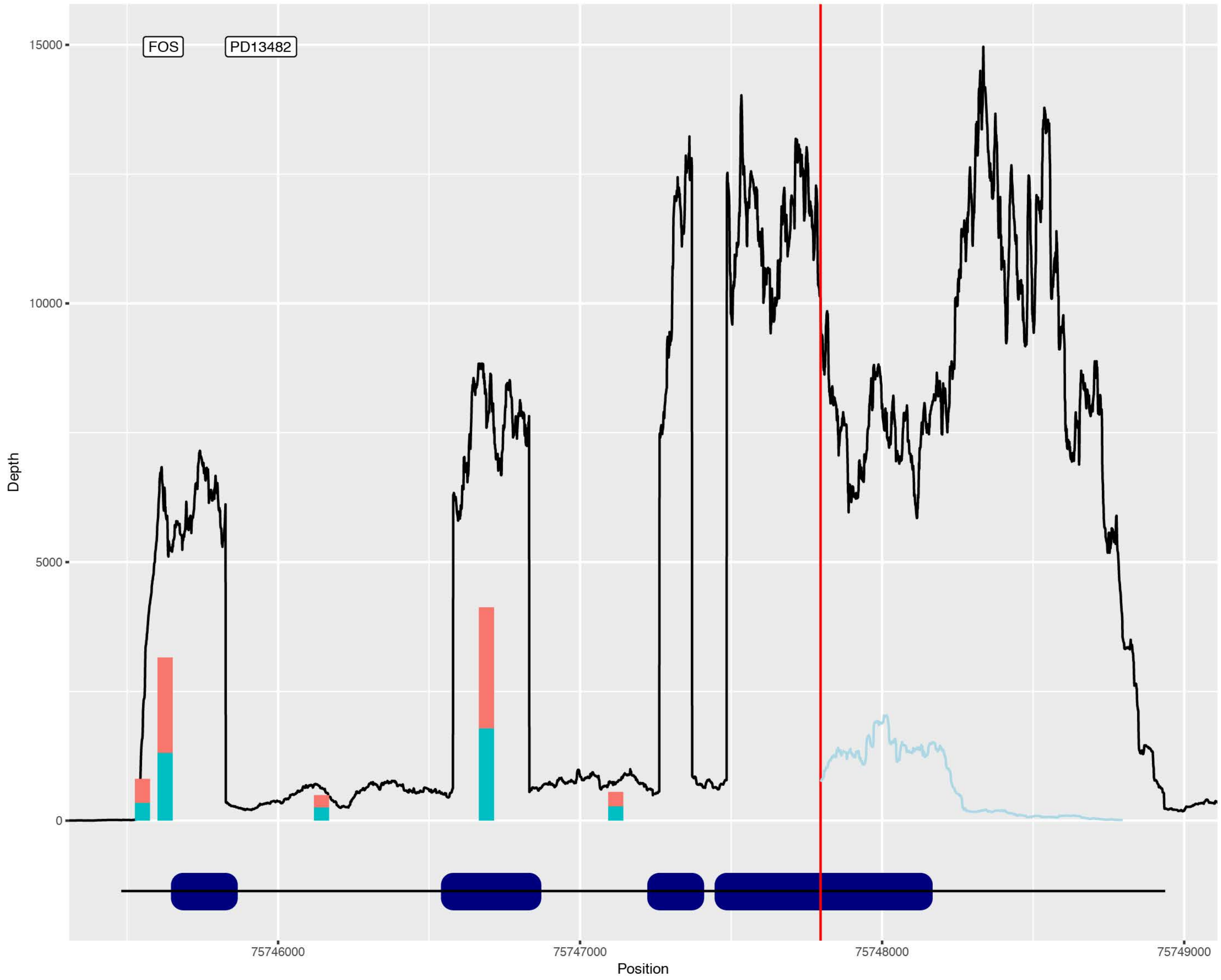

d

PD7519 (FOS+ve)

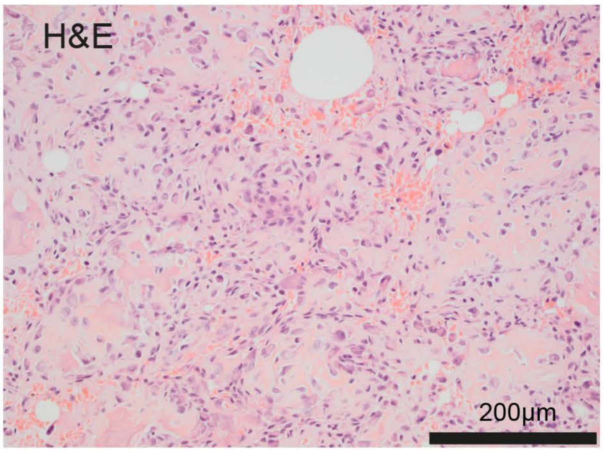

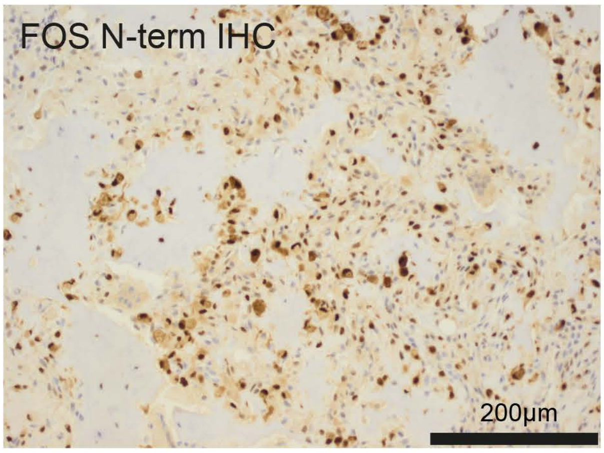

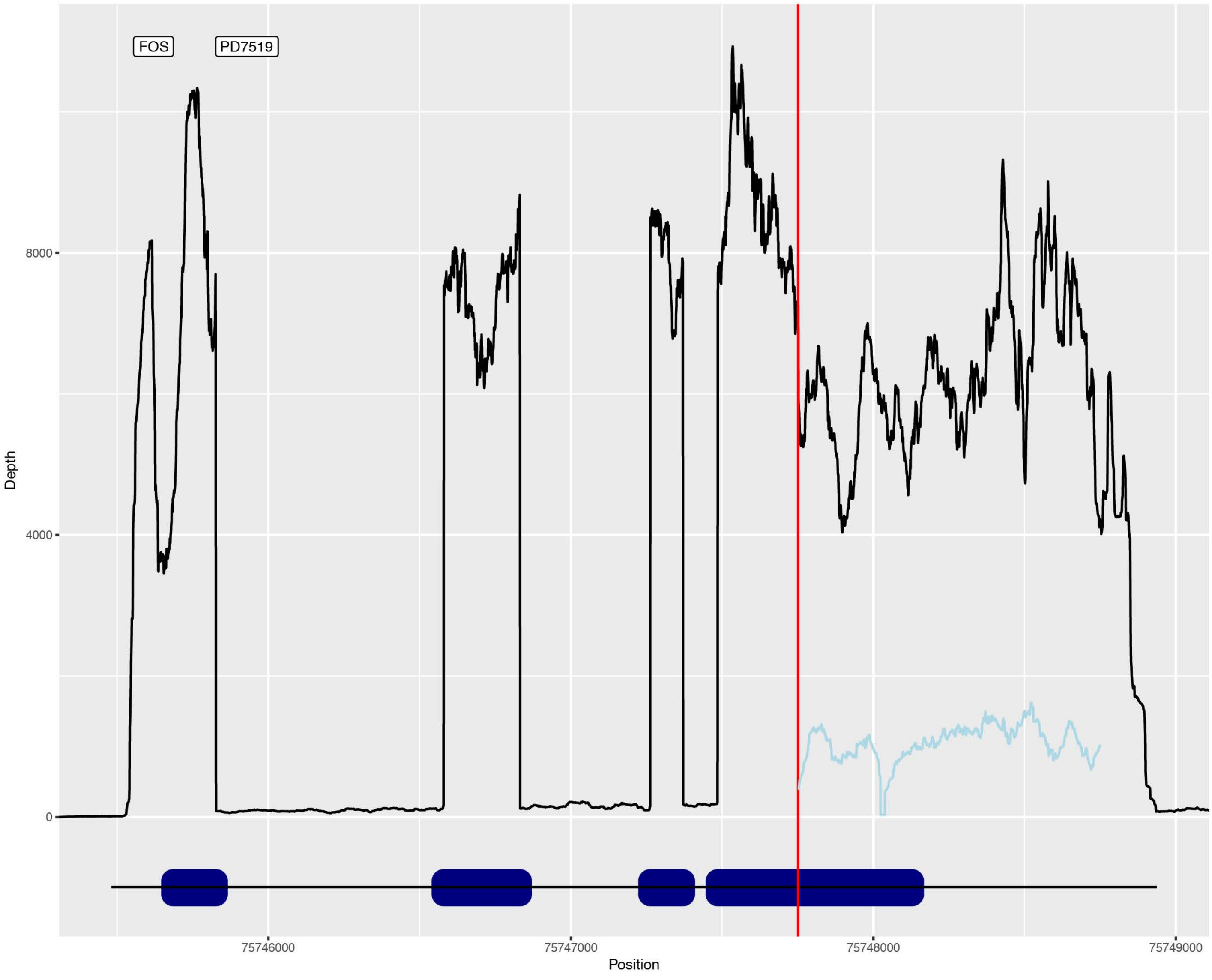

e

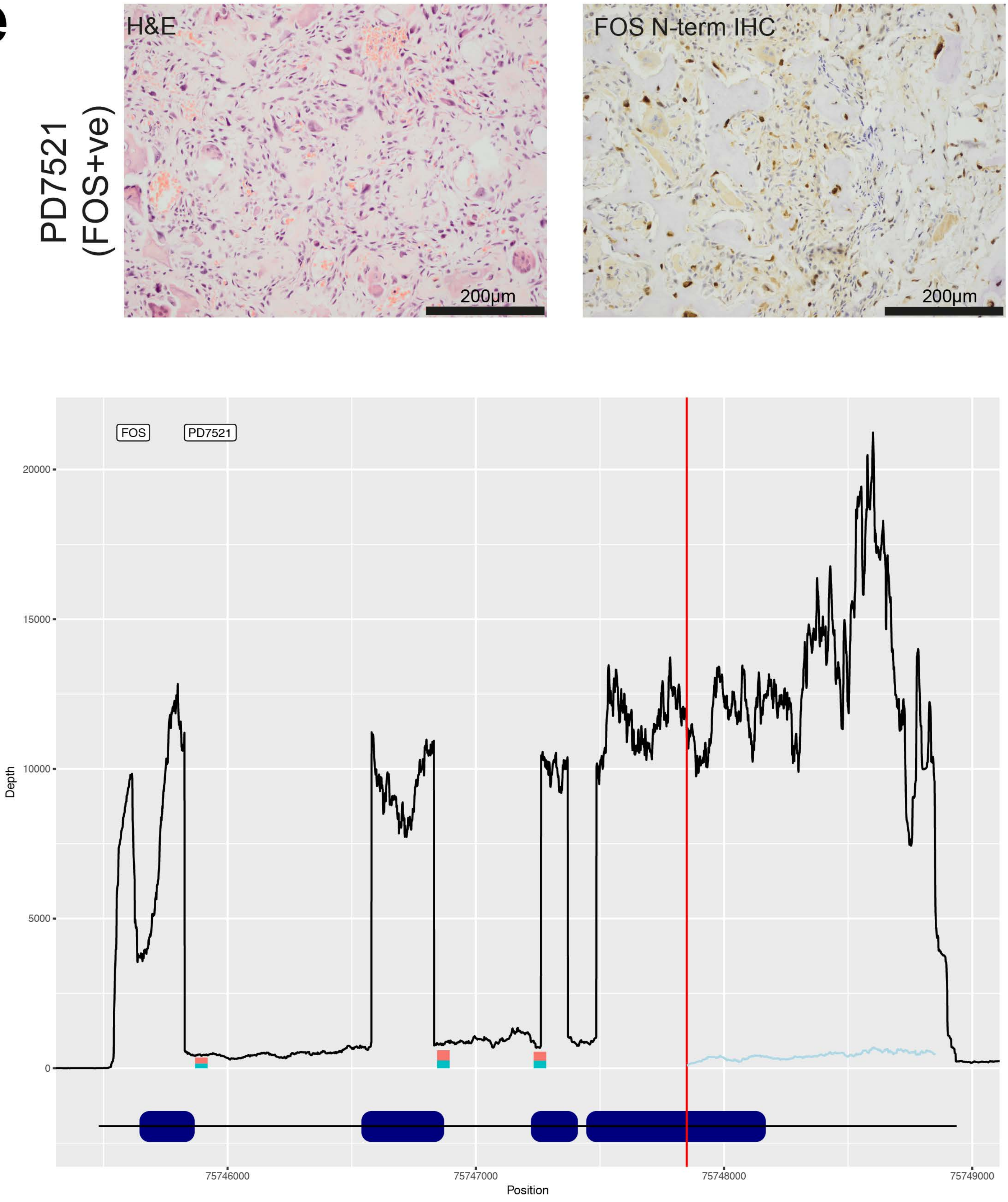
PD7521
(FOS+ve)
H&E
200µm
FOS N-term IHC
200µm
FOS
PD7521
20000
15000
10000
5000
0
Depth
75746000
75747000
75748000
75749000
Position

f

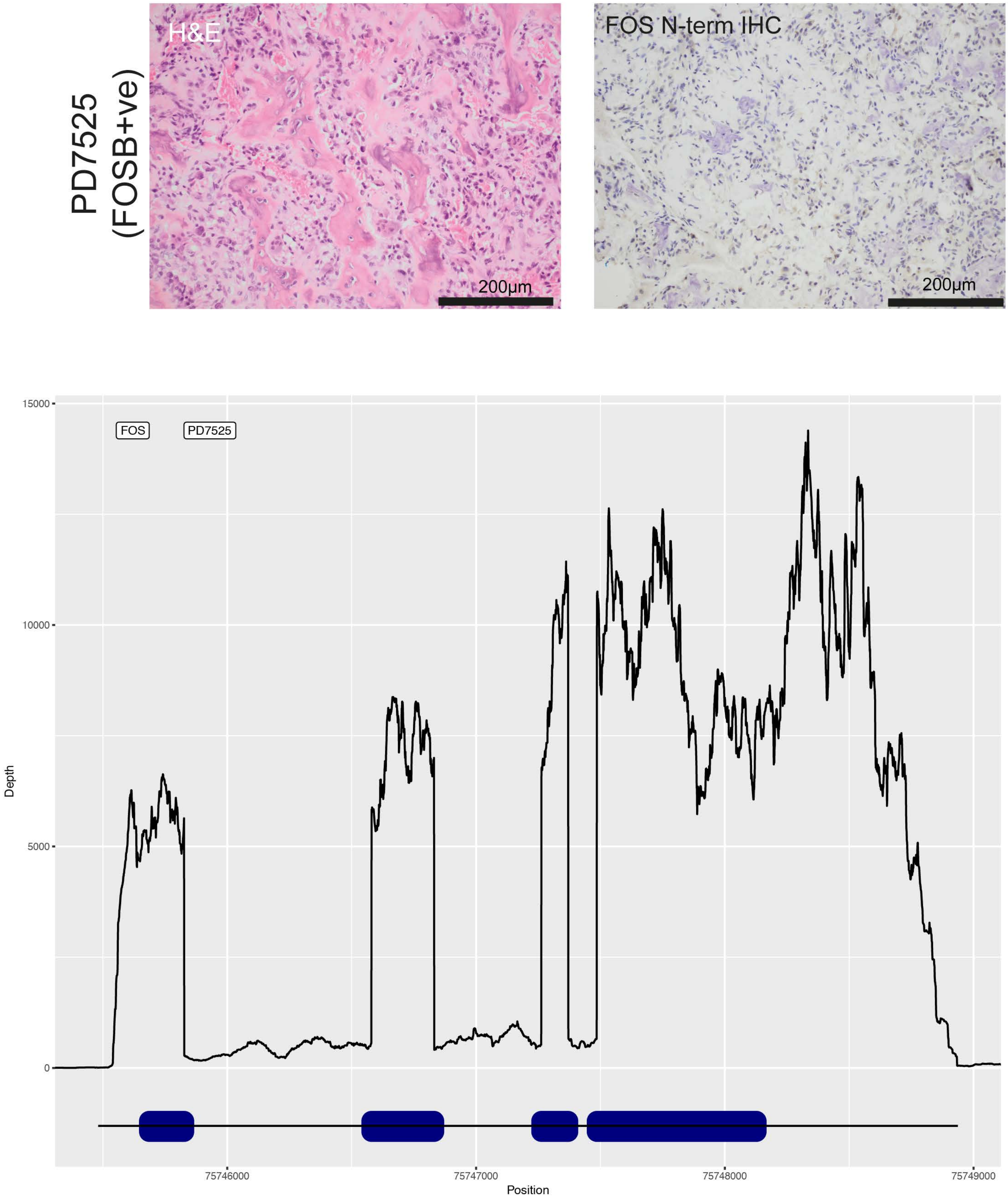
PD7525
(FOSB+ve)
H&E
200µm
FOS N-term IHC
200µm
FOS
PD7525
15000
10000
5000
0
Depth
75746000
75747000
75748000
75749000
Position

g

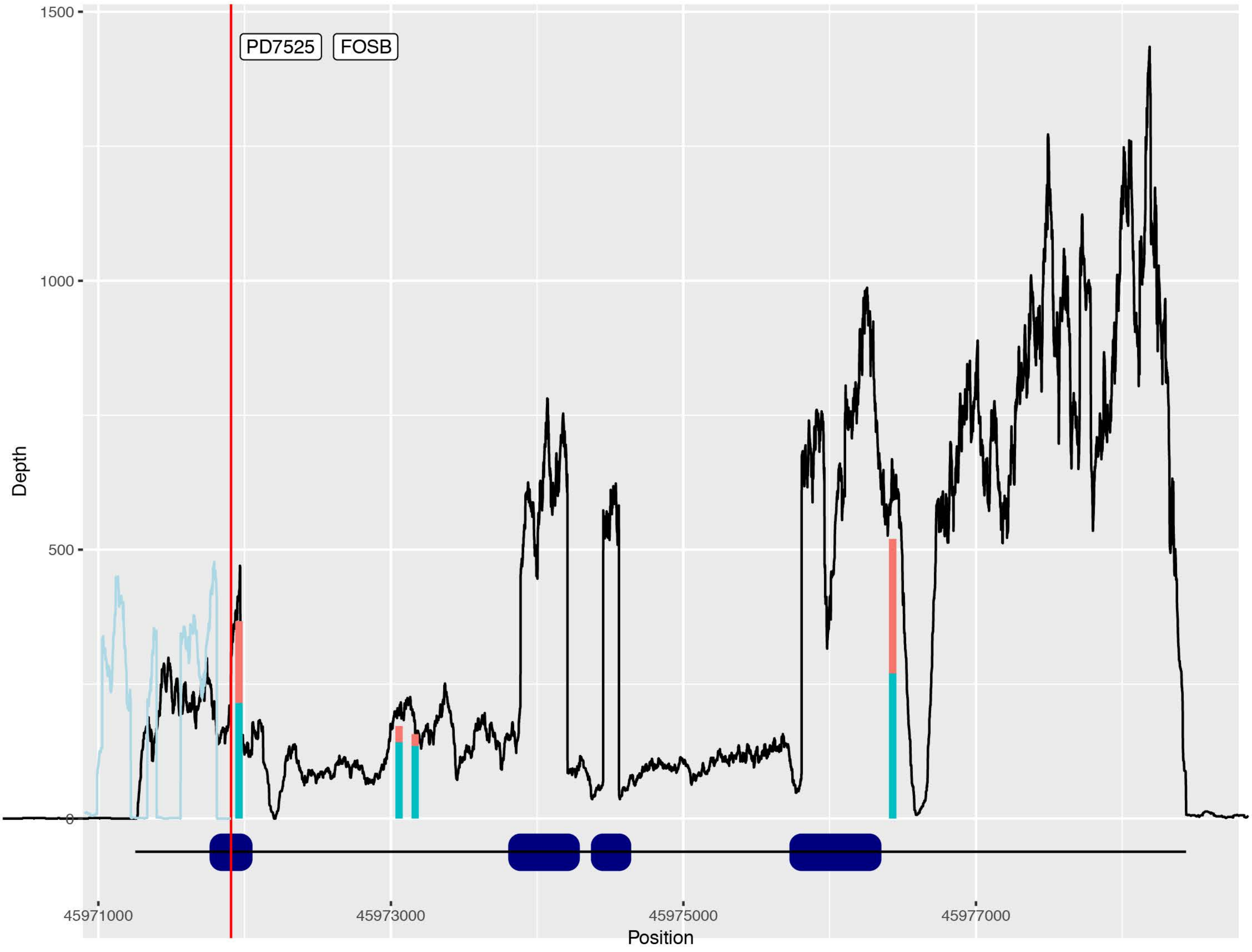

PD7525
FOSB
1500
1000
500
0
Depth
45971000
45973000
45975000
45977000
Position

Supplementary Figure 4. Validation of immunohistochemistry and additional samples
a) Demonstration of stronger nuclear reactivity of the N-terminal FOS antibody than the colonic epithelium positive control, even at the lowest concentration. H&E appearances for this sample are typical for osteoblastoma whilst FISH demonstrates a clear FOS break apart. b) Strong N-terminal FOS immunoreactivity is seen in osteoblastoma cases even for which breakpart signal could not be demonstrated by FISH. c) FOS or FOSB immunostaining was not seen in PD7525 likely owing to decalcification. PD7525 has a proven FOSB breakapart by DNA and RNA sequencing and FISH. FOSB antibody stains a pseudomyogenic haemangioendothelioma positive control well. d) The single osteosarcoma sample that demonstrated strong FOS immunoreactivity, demonstrated a distinct histological pattern and evidence of no FOS breakapart, though there is evidence of an amplification near the locus of FOS.

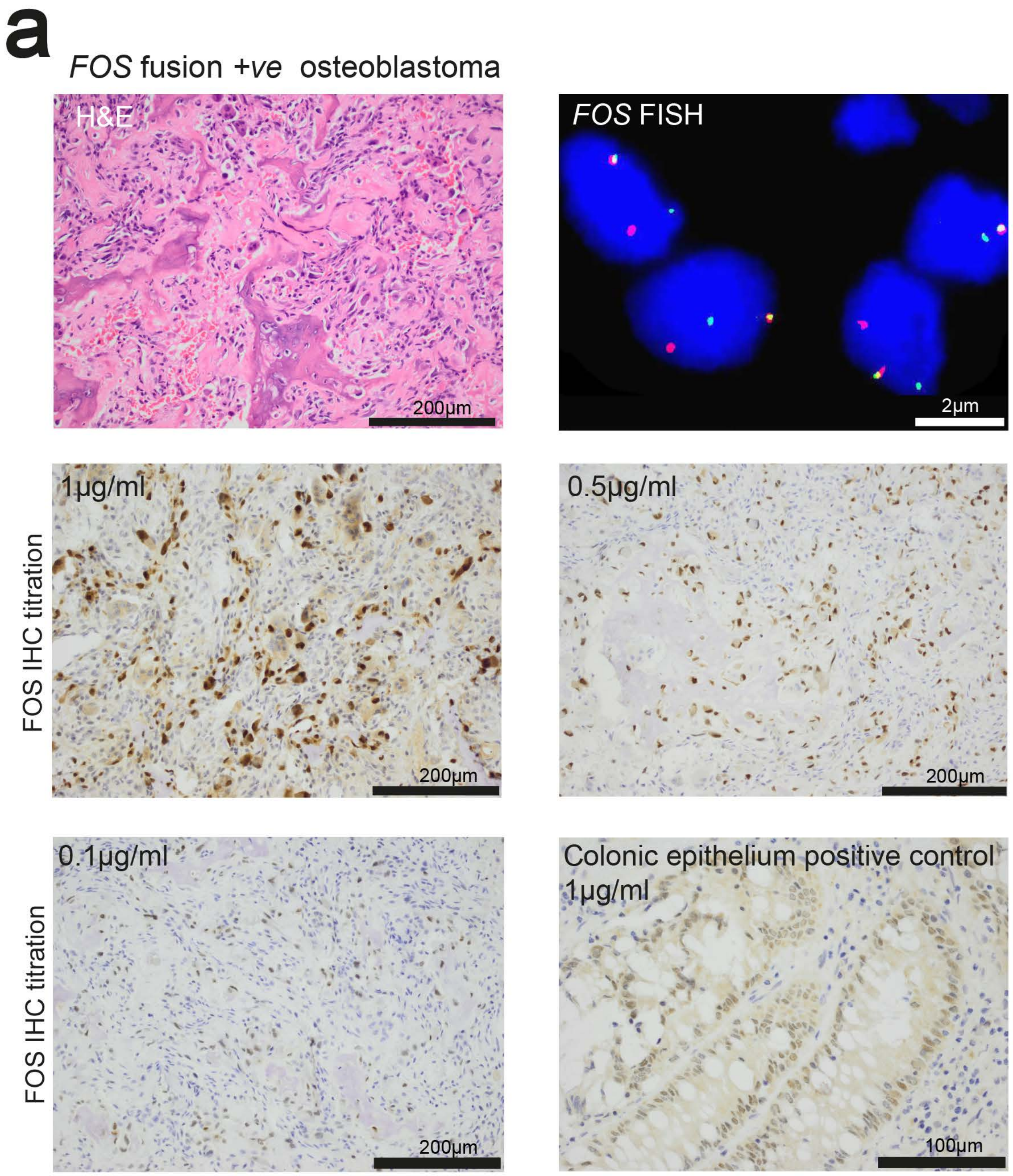

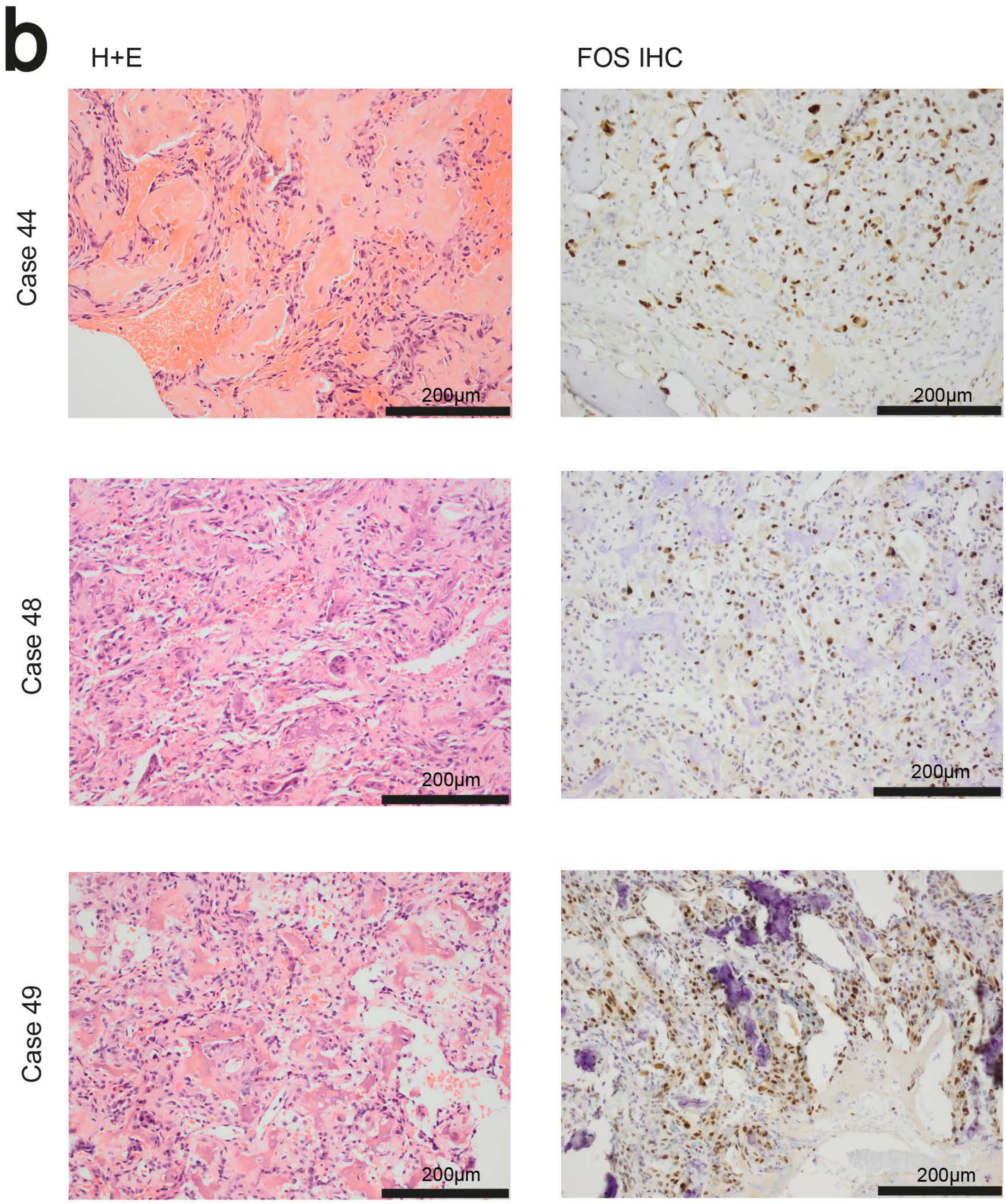
b
H+E
FOS IHC
Case 44
Case 48
Case 49
200µm
200µm
200µm
200µm
200µm
200µm

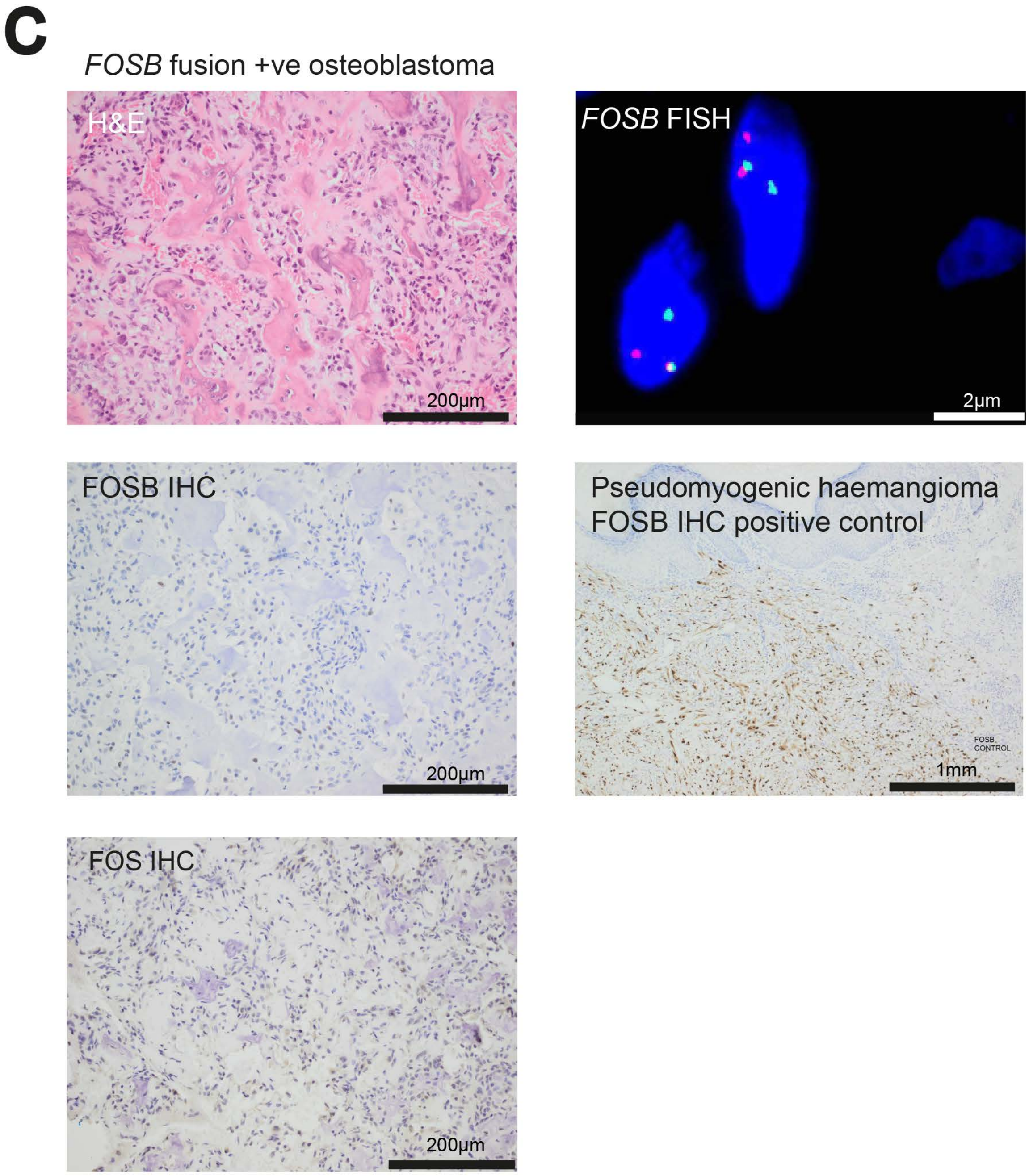
C
FOSB fusion +ve osteoblastoma
H&E
200µm
FOSB FISH
2µm
FOSB IHC
200µm
Pseudomyogenic haemangioma
FOSB IHC positive control
1mm
FOS IHC
200µm

d

Osteosarcoma

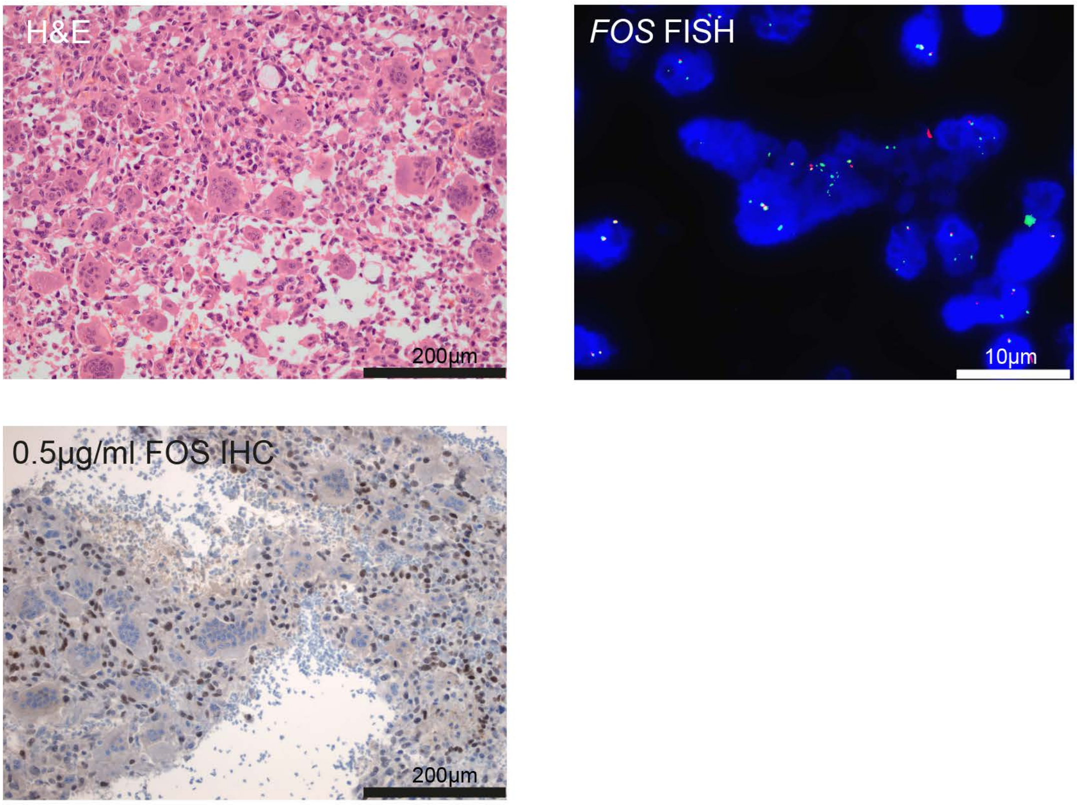

Supplement: Supplementary file 1 — Supplementary Information [file 41467_2018_4530_MOESM1_ESM.pdf]
