# Supplementary material for: Recurrent rearrangements of FOS and FOSB define osteoblastoma
Source: Nat Commun. 2018 Jun 1;9:2150. doi: 10.1038/s41467-018-04530-z (PMC5984627; doi:10.1038/s41467-018-04530-z)
Supplement: Supplementary file 3 — Description of Additional Supplementary Files [file 41467_2018_4530_MOESM3_ESM.pdf]

Description of Additional Supplementary Files:

Supplementary Data 1: A summary of clinical characteristics, sequencing, immunohistochemistry and FISH findings in all assessed osteoblastoma samples.

Supplementary Data 2: All substitutions and indels in 6 osteoblastoma genomes.

Supplementary Data 3: Substitutions and indels in 6 osteoblastoma genomes within genes.

Supplementary Data 4: Genome wide copy number changes of 6 osteoblastomas.

Supplementary Data 5: Structural variants in 6 osteoblastoma genomes.

Supplementary Data 6: DNA discordant reads spanning FOS and FOSB breakpoints, as appropriate to the sample. NB No matching Normal reads detected.

Supplementary Data 7: DNA split reads across the detected breakpoints in FOS, FOSB and their partners.

Supplementary Data 8: cDNA potentially split reads around detected FOS and FOSB breakpoints and their rearrangement partners.

Supplementary Data 9: Alignment QC and library statistics.

Supplementary Data 10: Amplificaiton and Sanger sequencing primers for cDNA validation of FOS fusions.

Supplementary Data 11: FISH BAC Clones.
